# Accurate Classification of Chunmee Tea Grade Using NIR Spectroscopy and Fuzzy Maximum Uncertainty Linear Discriminant Analysis

**DOI:** 10.3390/foods12030541

**Published:** 2023-01-26

**Authors:** Xiaohong Wu, Fei He, Bin Wu, Shupeng Zeng, Chengyu He

**Affiliations:** 1 School of Electrical and Information Engineering, Jiangsu University, Zhenjiang 212013, China; 2 High-Tech Key Laboratory of Agricultural Equipment and Intelligence of Jiangsu Province, Jiangsu University, Zhenjiang 212013, China; 3 Department of Information Engineering, Chuzhou Polytechnic, Chuzhou 239000, China

**Keywords:** Chunmee tea, tea grade, near-infrared spectra, feature extraction, standard normal variable, maximum uncertainty linear discriminant analysis

## Abstract

The grade of tea is closely related to tea quality, so the identification of tea grade is an important task. In order to improve the identification capability of the tea grade system, a fuzzy maximum uncertainty linear discriminant analysis (FMLDA) methodology was proposed based on maximum uncertainty linear discriminant analysis (MLDA). Based on FMLDA, a tea grade recognition system was established for the grade recognition of Chunmee tea. The process of this system is as follows: firstly, the near-infrared (NIR) spectra of Chunmee tea were collected using a Fourier transform NIR spectrometer. Next, the spectra were preprocessed using standard normal variables (SNV). Then, direct linear discriminant analysis (DLDA), maximum uncertainty linear discriminant analysis (MLDA), and FMLDA were used for feature extraction of the spectra, respectively. Finally, the *k*-nearest neighbor (KNN) classifier was applied to classify the spectra. The *k* in KNN and the fuzzy coefficient, *m*, were discussed in the experiment. The experimental results showed that when *k* = 1 and *m* = 2.7 or 2.8, the accuracy of the FMLDA could reach 98.15%, which was better than the other two feature extraction methods. Therefore, FMLDA combined with NIR technology is an effective method in the identification of tea grade.

## 1. Introduction

Chunmee tea belongs to green tea, which has many beneficial effects on the human body, such as anti-aging, anti-bacterial, lowering blood fat, weight loss, and so on [1,2,3]. Different grades of Chunmee tea have certain differences in price and flavor. In addition, there are cases of adulteration and the sale of substandard tea in the tea market [4]. Therefore, it is of practical significance to study and design an effective tea grade identification system.

At present, the identification methods of tea grade are mainly based on human sensory evaluation and chemical detection. Human sensory evaluation is an ancient method of tea grade identification. Human sensory evaluation is the identification of tea quality based on the senses of the evaluator, but it takes a lot of time and money to train an experienced evaluator [5,6]. Chemical detection is another common method for tea grade identification. Due to the complicated steps and high cost, the chemical detection method is not suitable for large-scale tea grade detection [7,8]. In addition, there are other ways to identify tea quality, such as electronic nose technology [9,10], hyperspectral technology [6,11], and computer vision technology [12,13]. Compared with other methods, the near-infrared (NIR) method for tea grade classification has the advantages of simple operation, low cost, and short time consumption. NIR spectroscopy is widely used in food, agriculture, chemistry, and other fields [14,15]. Ren et al. made use of the support vector machine (SVM) based on the linear kernel function to classify the NIR spectra of Keemun black tea and obtained the ideal classification results [16]. Supervised orthogonal locality preserving projection (SOLPP) was used to extract the features of NIR spectra, achieving the accurate identification of the grade, variety, and origin of green tea [17]. Chen et al. combined a random forest algorithm with NIR technology to establish an effective tea quality classification model [18].

NIR spectra have the characteristics of high dimension, redundancy, and overlap, which restrict the accuracy of subsequent classification [19]. The commonly used qualitative or quantitative analysis process of NIR spectra is as follows: firstly, the spectra are pretreated, then the spectra are feature selected or feature extracted, and finally, the classification or regression operation is carried out. Feature extraction is very important for accurate classification. When the sample dimension is larger than the number of samples, the small sample size (SSS) problem will occur [20]. The high dimensional characteristics of NIR data are often accompanied by the SSS problem. To solve the SSS problem, researchers have proposed many methods [21,22,23,24,25,26], which include direct linear discriminant analysis (DLDA) and maximum uncertainty linear discriminant analysis (MLDA). In the process of principal component analysis (PCA) dimension reduction, some discriminant information may be discarded, while the method of extracting features directly from high-dimensional data avoids this problem [27]. DLDA can be directly used for the feature extraction of high-dimensional data without PCA for dimensional reduction [25,28]. MLDA can be used for the feature extraction of high-dimensional data either directly or after dimension reduction [26]. But these two methods assume that each data point belongs strictly to one category or another, which is sometimes not appropriate. The fuzzy set theory, proposed by Zadeh, can well solve the problem of samples being difficult to partition [29]. At present, the feature extraction algorithm based on the fuzzy idea is applied to the extraction of spectral information. Fuzzy improved linear discriminant analysis (FILDA) has extracted the characteristics of the NIR spectra of the red jujube and has realized the effective classification of five kinds of red jujube [30]. Fuzzy discriminant principal component analysis (FDPCA) has extracted the characteristics of liquor, which has realized the accurate classification of liquor [31]. Fuzzy uncorrelated discriminant transform (FUDT) extracted discriminant information from the NIR spectra of milk samples and could achieve higher classification performance than uncorrelated discriminant transform (UDT) and linear discriminant analysis (LDA) [32]. Therefore, it is feasible to extract discriminant information from NIR spectra based on fuzzy feature extraction methods.

## 2. Materials and Methods

### 2.1. Sample Preparations

The experimental sample belongs to Chunmee tea, which is a variety of Mee tea. According to the Chinese National Standard GB/T 14456.5-2016 (Geographical indication products), Chunmee tea samples are divided into seven grades: Chunmee super grade, super grade one, super grade two, Chunmee grade one, Chunmee grade two, Chunmee grade three, and Chunmee grade four. Since only the first six grades of Chunmee tea were purchased, the experiment only measured the spectra of the first six grades of Chunmee tea. The sample preparation process was as follows: firstly, an electronic balance was used to weigh 3.0 g tea samples, and the weighed tea samples were put into the beaker. Secondly, 150 mL of 10 °C hot water was poured into the beaker. When the tea soup was cooled to room temperature, filter paper and a funnel were used to filter the tea dregs, and the tea soup was retained. At last, the pipette gun was used to suck a small amount of the tea soup into a quartz dish, and the tea soup is measured with a NIR spectrometer. During the experiment, the temperature was 20 °C, and the indoor humidity remained constant. Sixty tea samples were prepared for each grade, with a total of three hundred and sixty tea samples.

### 2.2. Collection of Spectra

The Antaris II Fourier transform NIR spectrometer (Thermo Fisher Scientific Co., Waltham, MA, USA) was applied to collect the NIR spectra of Chunmee tea samples during the experiment. Firstly, it was necessary to turn on the Antaris II spectrometer and preheat it for 1 h before starting to collect spectra. Secondly, the spectral range, sampling frequency, sampling times, and scanning interval of the spectrometer were set to 10,000–4000 cm^−1^, 4 cm^−1^, 32, and 3.857 cm^−1^, respectively, for the acquisition process. Finally, each tea sample was sampled three times, and its average value was taken as the final experimental data, which are 1557-dimensional NIR spectra data. In the experiment, 70% of the samples of each class were randomly selected as the training sample set and the remaining 30% as the test samples. As a result, there were 252 training samples and 108 test samples [33]. The experimental software was Matlab (The Mathworks Inc., Natick, MA, USA) 2015b.

### 2.3. Grade Identification System

**Figure 1:**
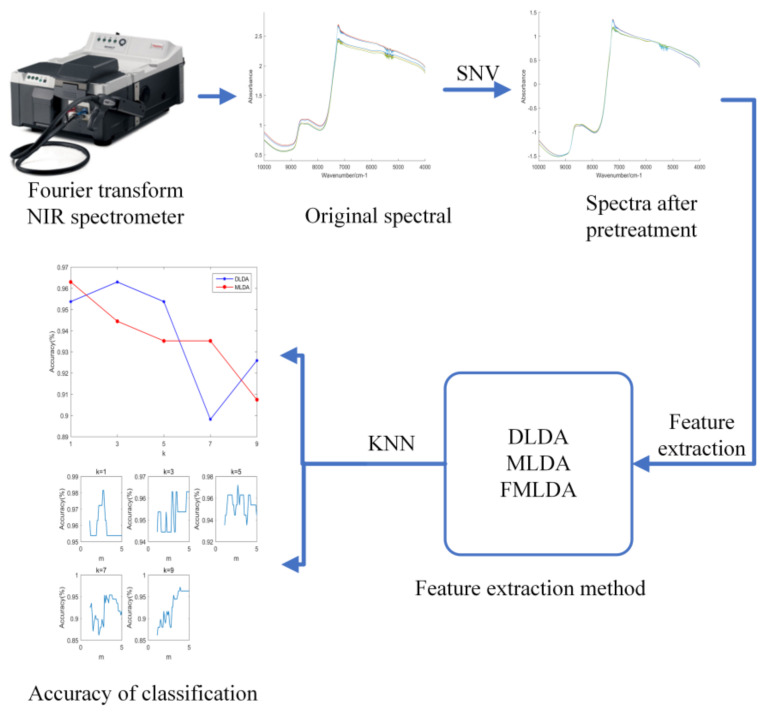
Flow chart of tea grade identification system based on feature extraction methods.

In this paper, fuzzy maximum uncertainty linear discriminant analysis (FMLDA) was proposed and applied to the identification of Chunmee tea grade. According to this method, a tea-grade identification system was established. As is shown in Figure 1, the tea grade system is constructed in the arrow direction. First, a Fourier transform NIR spectrometer was utilized to collect the NIR spectra of Chunmee tea, and then SNV was used to pretreat the spectra. Then DLDA, MLDA, and FMLDA were made for feature extraction for the spectral feature information, respectively. Finally, the *k*-nearest neighbor (KNN) classifier was used for classification. The results show that the proposed FMLDA method had a high classification accuracy. Therefore, the combination of FMLDA and NIR technology was effective in the recognition of Chunmee tea grade.

### 2.4. Maximum Uncertainty Linear Discriminant Analysis

Lukic et al. argued that the choice of the number of principal components of PCA affects the accuracy of classification [34]. However, the selection of PCA’s principal components may lose some useful discriminative information. To solve this problem, Thomaz et al. suggested expanding the smaller eigenvalues of the within-class covariance matrix in LDA and keeping most of the larger eigenvalues unchanged, and this method was named MLDA [25]. MLDA uses the maximum entropy selection method to stabilize the with-class covariance matrix in multiples of the identity matrix [35]. This method overcomes the SSS problem and the instability of the LDA.

Given a dataset $X=\{x_{1},x_{2},\ldots ,x_{n}\}\in {ℜ}^{d}$ with *n* samples, the dataset can be divided into c classes. The within-class scattering matrix $S_{w}$ and between-class scattering matrix $S_{b}$ are given as follows:

(1) $$S_{w}=\sum_{i=1}^{c}\sum_{x_{k}\in c_{i}}(x_{k}-x_{i}){\left(x_{k}-x_{i}\right)}^{T}$$

(2) $$S_{b}=\sum_{i=1}^{c}n_{i}(x_{i}-\bar{x}){\left(x_{i}-\bar{x}\right)}^{T}$$

where $\bar{x}$ is the mean value of all samples, $x_{i}$ is the mean value of the ith class samples, and $n_{i}$ is the number of the ith class samples. The calculation process of MLDA is as follows (Algorithm 1): 

**Algorithm 1:** The steps of the MLDA algorithmStep 1: Calculate
$S_{w}$ and $S_{b}$ according to Equations (1) and (2);Step 2: Calculate the eigenvalue diagonal matrix _Λ_ and eigenvectors matrix *ϕ* of *S*_*p*_, *S*_*p*_ = *S*_*w*_/(*n* − *c*);Step 3: Calculate the eigenvalues *λ* and average eigenvalues
$\bar{\lambda }$ of *S*_*p*_;Step 4: Obtain the new eigenvalue diagonal matrix Λ* by replacing eigenvalues *λ* in Λ that are smaller than the average eigenvalue $\bar{\lambda }$ with $\bar{\lambda }$;Step 5: Calculate the modified within classscatter matrix $S_{w}^{*}=S_{p}^{*}(n-c)=(\phi \Lambda^{*}\phi^{T})(n-c)$, and then obtain the discriminant transformation vector by computing $S_{w}^{*}{}^{-1}S_{b}$.

### 2.5. Fuzzy Maximum Uncertainty Linear Discriminant Analysis

Linear discriminant analysis (LDA) classifies samples strictly into one class or another, and this method is considered a “hard classification” method [36]. However, this classification sometimes does not match the actual situation. As an improved method of LDA, MLDA also suffers from this problem. Fuzzy set theory uses membership to reflect the degree of correlation between samples and certain types of samples. Fuzzy set theory is a good solution to the problem of some samples being difficult to be classified. Therefore, in order to further extract the discriminant information in the overlapped NIR spectra, fuzzy set theory is introduced into MLDA in this paper, and fuzzy maximum uncertainty linear discriminant analysis is proposed.

The fuzzy c-means (FCM) method was used to obtain the clustering center $v_{i}$ and fuzzy membership $u_{ij}^{m},i=1,2,\ldots ,c$, and j=1,2,…,n, as shown in Equations (1) and (2). The fuzzy membership, $u_{ij}^{m}$, represents the membership degree of the *j*th sample belonging to the *i*th class.

(3) $$v_{i}=\frac{\sum_{j=1}^{n}u_{ij}^{m}x_{j}}{\sum_{j=1}^{n}u_{ij}^{m}}$$

(4) $$u_{ij}=\frac{1}{\sum_{k=1}^{c}{\left(\frac{\left||x_{j}-v_{i}|\right|}{\left||x_{j}-v_{k}|\right|}\right)}^{\frac{2}{m-1}}}$$

where *m* is the fuzzy coefficient; ||⋅|| is the Euclidean distance. The fuzzy within-class scattering matrix, $S_{fw}$, and fuzzy between-class scattering matrix, $S_{fb}$, are given as follows:

(5) $$S_{fw}=\sum_{i=1}^{c}\sum_{x_{k}\in c_{i}}(x_{k}-v_{i}){\left(x_{k}-v_{i}\right)}^{T}$$

(6) $$S_{fb}=\sum_{i=1}^{c}n_{i}(v_{i}-\bar{x}){\left(v_{i}-\bar{x}\right)}^{T}$$

FMLDA is described as follows (Algorithm 2):**Algorithm 2:** The steps of the FMLDA algorithmStep 1: Calculate *S*_*fw*_ and *S*_*fb*_ according to Equations (5) and (6);Step 2: Calculate the eigenvalue diagonal matrix Λ and eigenvectors matrix *ϕ* of *S*_*fp*_, *S*_*fp*_ = *S*_*fw*_/(*n* − *c*);Step 3: Calculate *λ*, $\bar{\lambda }$, and Λ* using the same as the process of MLDA;Step 4: Calculate the modified fuzzy within-class scatter matrix $S_{fw}^{*}=S_{fp}^{*}(n-c)=(\phi \Lambda^{*}\phi^{T})(n-c)$, and then obtain the discriminant transformation vector by computing $S_{fw}^{*}{}^{-1}S_{fb}$.

### 2.6. K-Nearest Neighbor

KNN is one of the commonly used machine learning algorithms, which can be used for classification and regression. As a supervised learning algorithm, KNN is implemented as follows: First, the distance between the object to be classified and other objects is calculated. Then, the nearest *k* neighbors are counted. Finally, for the *k* nearest neighbors, the object to be classified is categorized based on which category most of them belong to. It can be seen that the performance of KNN is strongly influenced by the parameter *k*.

## 3. Results

### 3.1. Spectral Analysis and Pretreatment

**Figure 2:**
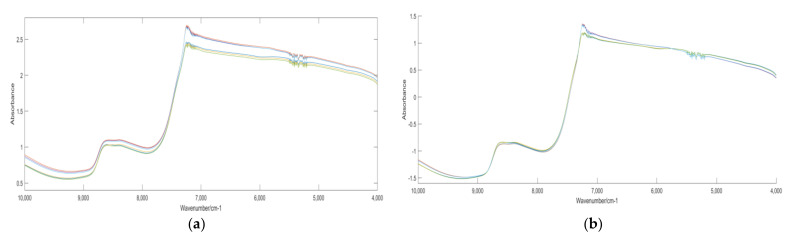
Original spectra and pretreated spectra: (**a**) Original mean NIR spectra of Chunmee tea; (**b**) the mean spectra after SNV pretreatment.

The mean NIR spectra of Chunmee tea are shown in Figure 2a. It can be seen that the NIR spectra of different grades of Chunmee tea are very similar, and the absorption peak of Chunmee tea is between 7300 and 7100 cm^−1^. The absorption peak here is due to the stretching vibration absorption of hydroxyl (OH) and nitrogen–hydrogen (N–H) bonds in tea polysaccharides and hydrogen-containing compounds [37]. In addition, there are a large number of burrs in the spectrum between the wavelengths 5500 and 5100 cm^−1^, which may be due to the noise introduced during the experiment. The original NIR spectra of Chunmee tea have noise and scattering, which need to be treated. The standard normal variable (SNV) is a preprocessing method for spectral scattering [38], and the spectra were preprocessed by the SNV in the experiment. Figure 2b shows the mean spectra of Chunmee tea pretreated with the SNV. It can be seen that, after pretreatment, the noise in the 5500–5100 cm^−1^ region was reduced, and the spectra scattering was also treated to a certain extent.

### 3.2. Feature Extraction Using DLDA, MLDA, and FMLDA

DLDA is a direct feature extraction method for high-dimensional data. The core of the DLDA algorithm is to find a matrix *A* that can make the between-class scattering matrix and with-class scattering matrix diagonalized at the same time. The DLDA process is as follows: At first, it is necessary to find a matrix, Y, such that it satisfies $Y^{T}S_{b}Y=D_{b}$, where $Y^{T}Y=I$, $D_{b}$ is the diagonal matrix in descending order. Then, let $Z=YD_{b}^{-1/2}$ diagonalize $Z^{T}S_{w}Z$ and decompose the eigenvalue $Z^{T}S_{w}Z$ to obtain the eigenvalue matrix, $D_{w}$, and the eigenvector matrix, U. Finally, let $A=U^{T}Z^{T}$; then, the diagonalization of $S_{b}$ and $S_{w}$ is achieved simultaneously. Then, the transformation matrix of DLDA is $W=D_{w}^{-1/2}A$. The dimensionality of the tea sample is still 1557 dimensions after pre-processing. The distribution of tea samples at this point is more chaotic, and in order to obtain a high accuracy rate for the subsequent tea class classification, DLDA is used to extract features from the pre-processed data. The number of discriminant vectors of DLDA was set to 5 in the experiment.

MLDA can also be directly used for the feature extraction of high-dimensional data to avoid the loss of discriminant information in the process of dimension reduction. In the experiment, the discriminant vector number of MLDA was set to 5. The data after SNV processing was mapped to the low-dimensional space through the discriminant vectors of MLDA, and the data were classified by the classifier in the low-dimensional space. The size of the test set after MLDA mapping was 108 × 5. That is, 108 was the number of test sets, and 5 was the dimension of the test set after discriminant vector mapping.

FMLDA can directly extract the features of the preprocessed high-dimensional data. Before running FMLDA, it is necessary to obtain the final cluster centers with FCM. The procedure is as follows: Firstly, the relevant parameters need to be set. The fuzzy coefficient, *m* > 1, and the number of categories, *c*, is set to 6. The iteration threshold, ε, and the maximum number of iterations, *r,* are set to 0.00001 and 100, respectively. The initial clustering center, $v^{\left(0\right)}$, is shown in Equation (7). The size of $v^{\left(0\right)}$ is 6 × 1557, where 6 denotes 6 grades of Chunmee tea and 1557 denotes the dimension of the Chunmee tea spectrum. Then the initial clustering center is substituted into Equation (4) to calculate the membership, $u_{ij}$, and then, $u_{ij}$ is utilized to calculate the new clustering centers with Equation (3). Finally, this iterative calculation is repeated until $||v_{i}^{\left(r+1\right)}-v_{i}^{\left(r\right)}||<\varepsilon$ or *r* > 100 is satisfied and the iteration ends [39]. The discriminant vector number of FMLDA is set to be the same as that of MLDA. After the projection of FMLDA, the test set data is converted to the subspace composed of discriminant vectors of FMLDA. At this time, the test set data size is 108 × 5, where 108 and 5 have the same meaning as in MLDA.

(7) $$v^{\left(0\right)}=\left[\begin{array}{l} v_{1}^{\left(0\right)}\\ v_{2}^{\left(0\right)}\\ v_{3}^{\left(0\right)}\\ v_{4}^{\left(0\right)}\\ v_{5}^{\left(0\right)}\\ v_{6}^{\left(0\right)} \end{array}\right]={\left[\begin{array}{l} 0.4106,0.4143,0.4145,\ldots ,-1.2362\\ 0.3493,0.3519,0.3504,\ldots ,-1.1608\\ 0.3955,0.3996,0.4002,\ldots ,-1.2417\\ 0.3522,0.3547,0.3531,\ldots ,-1.1759\\ 0.3983,0.4024,0.4026,\ldots ,-1.2353\\ 0.3597,0.3622,0.3606,\ldots ,-1.1791 \end{array}\right]}_{6\times 1557}$$

### 3.3. Classification Using KNN

KNN is a commonly used classification algorithm whose classification performance is strongly influenced by the parameter *k* [40]. The effects of different values of *k* on the extraction ability of DLDA, MLDA, and FMLDA were discussed. In the experiments, the value of *k* was set to an odd number between 1 and 9. The accuracy of FMLDA is also affected by the fuzzy coefficient, *m* [41]. In order to find the suitable *m*, set the range of *m* to 1~5 and the step size to 0.1.

**Figure 3:**
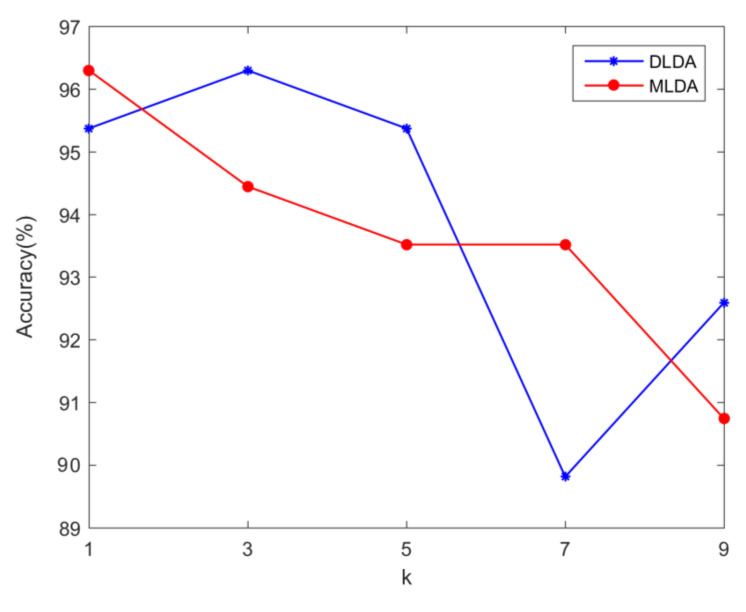
Accuracies of DLDA and MLDA at different *k* values.

**Figure 4:**
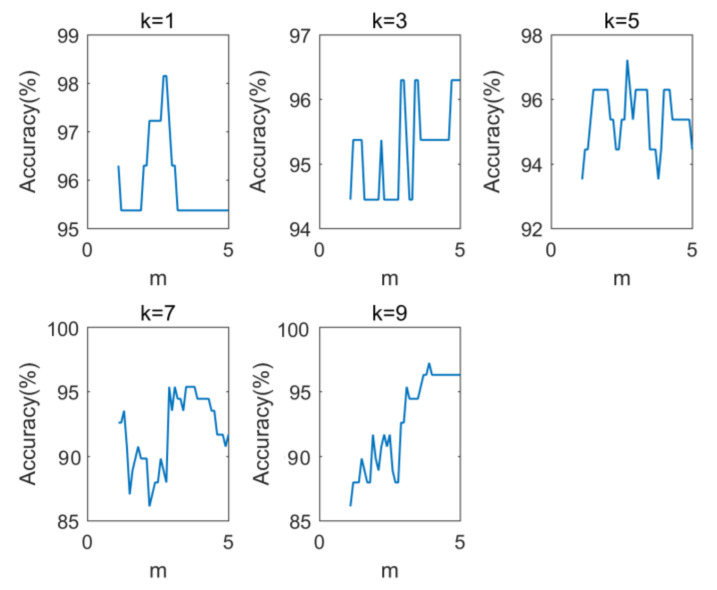
Accuracies of FMLDA under different *k* and *m* values.

The accuracies of DLDA and MLDA are shown in Figure 3. The accuracies of the two feature extraction methods basically decrease with the increase of the *k* value. The highest accuracy of DLDA and MLDA was 96.3%, and the corresponding *k* was 3 and 1, respectively. Both DLDA and MLDA correctly identified 104 test sets. The operation result of FMLDA is shown in Figure 4. There are 5 subgraphs in the figure, representing the accuracy rate of FMLDA under different combinations of *k* and *m*. When *k* = 1, the accuracy of FMLDA first increases and then decreases with the increase of *m*. When *m* = 2.7 and 2.8, FMLDA can achieve the highest accuracy level of 98.15%, and 106 test sets can be accurately identified. When *k* is 3, 5, 7, and 9, the accuracies of FMLDA show an oscillating trend, and the highest accuracy levels can be achieved as 96.3%, 97.22%, 95.37%, and 97.22%, respectively. It can be seen that under different *k* values, the highest accuracy of FMLDA is above 95%, while DLDA and MLDA decrease to below 95% with the increase of the *k* value. This indicates that the accuracy rate of FMLDA has higher stability under different *k* values.

## 4. Discussion

In this study, in order to further extract the feature information of NIR spectra, the FMLDA algorithm is proposed. FMLDA was used to extract the spectral features of Chunmee tea, and a tea grade identification system based on NIR spectroscopy was established. The system first used an Antaris II spectrometer to collect tea NIR spectra. The spectra were then pretreated with the SNV. In addition, DLDA and MLDA were also used for feature extraction. Finally, KNN was used for classification. Table 1 records the highest accuracy of the tea grade recognition model based on three feature extraction algorithms and the corresponding parameters. The correct number indicates the number of correctly identified test samples. Both DLDA and MLDA models can achieve an accuracy rate of 96.3%. The accuracy rate of the model based on FMLDA is 98.15%, which is 1.85% higher than that of the other two models. The tea grade recognition model based on DLDA and MLDA misclassified two Chunmee super grade samples, one Chunmee grade one sample, and one Chunmee grade three sample in the test set. The model based on FMLDA only misclassified two Chunmee super-grade samples, and the rest of the samples were correctly classified. It can be considered that, compared with the other two feature extraction methods, FMLDA can better identify some indistinguishable data, so as to obtain more feature information and further improve the accuracy of the tea grade recognition model.

In order to further investigate the effect of the division ratio between the training set and the test set on the feature extraction results, the training set, and the test set were divided according to 5:5 (180/180) and 6:4 (216/144) for the experiments. The experimental results are shown in Table 2. As can be seen from Table 2, at the ratios of 5:5 and 6:4, the accuracy of the tea grade recognition model based on FMLDA is better than that of DLDA and MLDA. This also indicates that, in the stage of extracting tea spectral characteristics, FMLDA obtains more characteristic information. Therefore, the FMLDA method based on fuzzy set theory is effective for the feature extraction of NIR spectra. In conclusion, FMLDA combined with SNV and KNN can identify Chunmee tea grade accurately.

## 5. Conclusions

This paper proposes a feature extraction method, called FMLDA, for the feature extraction of the NIR spectra of Chunmee tea. A tea grade identification system is established using this method. The process of this system was as follows: Firstly, the NIR spectra of Chunmee tea were collected using a Fourier transform NIR spectrometer. Then, the SNV was applied to pretreat the spectra, and then FMLDA was used to extract the features of the pretreated spectra. During this period, FMLDA was compared with two other feature extraction methods (DLDA and MLDA). Finally, the spectra, after feature extraction, were classified using KNN. The results show that the tea grade model based on the feature extraction method of FMLDA has a higher accuracy than the other two models, and FMLDA can obtain the NIR spectral characteristics of Chunmee tea more effectively. The combination of FMLDA and NIR spectroscopy is an effective tea-grade identification model, and SNV-FMLDA-KNN has a good effect on the tea grade recognition system. 

## Figures and Tables

**Table 1:** Accuracies of tea grade recognition system based on three feature extraction methods.

Model	*k*	*m*	Correct Number	Accuracy (%)
SNV-DLDA-KNN	3	-	104	96.3
SNV-MLDA-KNN	1	-	104	96.3
SNV-FMLDA-KNN	1	2.7, 2.8	106	98.15

**Table 2:** Accuracies of tea grade recognition system under different numbers of training/test samples.

Training/Test Samples	Model	Accuracy (%)
180/180	SNV-DLDA-KNN	92.78
	SNV-MLDA-KNN	92.78
	SNV-FMLDA-KNN	93.33
216/144	SNV-DLDA-KNN	94.44
	SNV-MLDA-KNN	94.44
	SNV-FMLDA-KNN	95.14

## Data Availability

The data are available from the corresponding author.

## References

[1] Chen Y., Cheng S., Dai J., Wang L., Xu Y., Peng X., Xie X., Peng C. (2021). Molecular mechanisms and applications of tea polyphenols: A narrative review. J. Food Biochem..

[2] Mansoori R., Jain D., Pandey V., Jain S.K. (2022). A comprehensive review on biological activity of green tea (Camellia sinensis). J. Drug Deliv. Ther..

[3] Zhang Z.Y., Liu C.W., Fang W.W., Tang Q.Q., Zhan L., Shi Y., Tang M.G., Liu Z.H., Zhang S., Liu A.L. (2022). Research progress on the lipid-lowering and weight loss effects of tea and the mechanism of its functional components. J. Nutr. Biochem..

[4] Cebi N., Yilmaz M.T., Sagdic O. (2017). A rapid ATR-FTIR spectroscopic method for detection of sibutramine adulteration in tea and coffee based on hierarchical cluster and principal component analyses. Food Chem..

[5] Ahmad H., Sun J., Nirere A., Shaheen N., Zhou X., Yao K. (2021). Classification of tea varieties based on fluorescence hyperspectral image technology and ABC-SVM algorithm. J. Food Process. Preserv..

[6] Li L.Q., Xie S.M., Ning J.M., Chen Q.S., Zhang Z.Z. (2019). Evaluating green tea quality based on multisensor data fusion combining hyperspectral imaging and olfactory visualization systems. J. Sci. Food Agric..

[7] Wang J., Wu X.H., Zheng J., Wu B. (2022). Rapid identification of green tea varieties based on FT-NIR spectroscopy and LDA/QR. Food Sci. Technol..

[8] Ding X.X., Ni Y.N., Kokot S. (2015). Analysis of different Flos Chrysanthemum tea samples with the use of two-dimensional chromatographic fingerprints, which were interpreted by different multivariate methods. Anal. Methods.

[9] Yu D., Gu Y. (2021). A machine learning method for the fine-grained classification of green tea with geographical indication using a MOS-based electronic nose. Foods.

[10] Yu H., Wang J. (2007). Discrimination of LongJing green-tea grade by electronic nose. Sens. Actuators B Chem..

[11] Liu C.L., Lu W.Y., Gao B.Y., Kimura H., Li Y.F., Wang J. (2020). Rapid identification of chrysanthemum teas by computer vision and deep learning. Food Sci. Nutr..

[12] Xu M., Wang J., Gu S. (2019). Rapid identification of tea quality by E-nose and computer vision combining with a synergetic data fusion strategy. J. Food Eng..

[13] Teye E., Huang X.Y., Afoakwa N. (2013). Review on the potential use of near infrared spectroscopy (NIRS) for the measurement of chemical residues in food. Am. J. Food Sci. Technol..

[14] Ozaki Y. (2012). Near-infrared spectroscopy-Its versatility in analytical chemistry. Anal. Sci..

[15] Ren G.X., Liu Y., Ning J.M., Zhang Z.Z. (2021). Assessing black tea quality based on visible–near infrared spectra and kernel-based methods. J. Food Compos. Anal..

[16] Liu P., Wen Y.P., Huang J.S., Xiong A.H., Wen J.P., Li H., Huang Y.F., Zhu X.Y., Ai S.R., Wu R.M. (2019). A novel strategy of near-infrared spectroscopy dimensionality reduction for discrimination of grades, varieties and origins of green tea. Vib. Spectrosc..

[17] Chen G.K., Zhang X.C., Wu Z.B., Su J.H., Cai G.R. (2021). An efficient tea quality classification algorithm based on near infrared spectroscopy and random Forest. J. Food Process Eng..

[18] Jiang D.Y., Qi G.Q., Hu G., Mazur N., Zhu Z.Q., Wang D. (2020). A residual neural network based method for the classification of tobacco cultivation regions using near-infrared spectroscopy sensors. Infrared Phys. Technol..

[19] Kong H., Wang L., Teoh E.K., Wang J.G., Venkateswarlu R. A framework of 2D Fisher discriminant analysis: Application to face recognition with small number of training samples. Proceedings of the 2005 IEEE Computer Society Conference on Computer Vision and Pattern Recognition (CVPR’05).

[20] Belhumeur P.N., Hespanha J.P., Kriegman (1997). Eigenfaces vs. Fisherfaces: Recognition using class specific linear projection. IEEE Trans. Pattern Anal. Mach. Intell..

[21] Ye J.P., Li Q. (2005). A two-stage linear discriminant analysis via QR decomposition. IEEE Trans. Pattern Anal. Mach. Intell..

[22] Ye J.P. (2005). Characterization of a family of algorithms for generalized discriminant analysis on undersampled problems. J. Mach. Learn Res..

[23] Lu J., Plataniotis K.N., Venetsanopoulos A.N. (2005). Regularization studies of linear discriminant analysis in small sample size scenarios with application to face recognition. Pattern Recognit. Lett..

[24] Yu H., Yang J. (2001). A direct LDA algorithm for high-dimensional data—with application to face recognition. Pattern Recognit..

[25] Thomaz C.E., Kitani E.C., Gillies D.F. (2006). A maximum uncertainty LDA-based approach for limited sample size problems—With application to face recognition. J. Braz. Comput. Soc..

[26] Juefei-Xu F., Savvides M. (2016). Multi-class Fukunaga Koontz discriminant analysis for enhanced face recognition. Pattern Recognit..

[27] Ueki K., Hayashida T., Kobayashi T. Subspace-based age-group classification using facial images under various lighting conditions. Proceedings of the 7th International Conference on Automatic Face and Gesture Recognition (FGR06).

[28] Zadeh L.A. (1965). Fuzzy sets. Inf. Control.

[29] Qi Z.X., Wu X.H., Yang Y.J., Wu B., Fu H.J. (2022). Discrimination of the red jujube varieties using a portable NIR spectrometer and fuzzy improved linear discriminant analysis. Foods.

[30] Wu X.H., Zhu J., Wu B., Zhao C., Sun J., Dai C.X. (2019). Discrimination of Chinese liquors based on electronic nose and fuzzy discriminant principal component analysis. Foods.

[31] Zhang T.F., Wu X.H., Wu B., Dai C.X., Fu H.J. (2022). Rapid authentication of the geographical origin of milk using portable near-infrared spectrometer and fuzzy uncorrelated discriminant transformation. J. Food Process Eng..

[32] Zhuang X.G., Shi X.S., Wang H.F., Wang L.L., Fang J.X. (2019). Rapid Determination of Green Tea Origins by Near-Infrared Spectroscopy and Multi-Wavelength Statistical Discriminant Analysis. J. Appl. Spectrosc..

[33] Sato J.R., Fujita A., Thomaz C.E., Martin M.D.G.M., Mourão-Miranda J., Brammer M.J., Junior E.A. (2009). Evaluating SVM and MLDA in the extraction of discriminant regions for mental state prediction. NeuroImage.

[34] Thomaz C.E., Gillies D.F., Feitosa R.Q. (2004). A new covariance estimate for Bayesian classifiers in biometric recognition. IEEE Trans. Circuits Syst. Video Technol..

[35] Setser A.L., Smith R.W. (2018). Comparison of variable selection methods prior to linear discriminant analysis classification of synthetic phenethylamines and tryptamines. Forensic Chem..

[36] Firmani P., Luca D.S., Bucci R., Marini F., Biancolillo A. (2019). Near infrared (NIR) spectroscopy-based classification for the authentication of Darjeeling black tea. Food Control.

[37] Tang N.Q., Sun J., Yao K.S., Zhou X., Tian Y., Cao Y., Adria N. (2021). Identification of Lycium barbarum varieties based on hyperspectral imaging technique and competitive adaptive reweighted sampling-whale optimization algorithm-support vector machine. J. Food Process Eng..

[38] Wu X.H., Wu B., Sun J., Yang N. (2019). Classification of apple varieties using near infrared reflectance spectroscopy and fuzzy discriminant c-means clustering model. J. Food Process Eng..

[39] Modaresi F., Araghinejad S. (2014). A comparative assessment of support vector machines, probabilistic neural networks, and K-nearest neighbor algorithms for water quality classification. Water Resour. Manag..

[40] Shen Y.J., Wu X.H., Wu B., Tan Y., Liu J.M. (2021). Qualitative analysis of lambda-cyhalothrin on Chinese cabbage using mid-infrared spectroscopy combined with fuzzy feature extraction algorithms. Agriculture.

[41] Mishra P., Nordon A., Tschannerl J., Lian G.P., Redfern S., Marshall S. (2018). Near-infrared hyperspectral imaging for non-destructive classification of commercial tea products. J. Food Eng..

